# Human and mouse angiogenins: Emerging insights and potential opportunities

**DOI:** 10.3389/fmicb.2022.1022945

**Published:** 2022-11-17

**Authors:** Mst. Farzana Sultana, Hirohito Abo, Hiroto Kawashima

**Affiliations:** ^1^Laboratory of Microbiology and Immunology, Graduate School of Pharmaceutical Sciences, Chiba University, Chiba, Japan; ^2^Department of Pharmacy, Jashore University of Science and Technology, Jashore, Bangladesh

**Keywords:** antimicrobial protein, angiogenin, angiogenesis, ribonuclease, gastrointestinal cancers

## Abstract

Angiogenin, a well-known angiogenic factor, is crucial to the angiogenesis in gastrointestinal tumors. Human angiogenin has only one gene, whereas the murine angiogenin family has extended to incorporate six genes. Evolutionary studies have suggested functional variations among murine angiogenin paralogs, even though the three-dimensional structures of angiogenin proteins are remarkably similar. In addition to angiogenesis, the ubiquitous pattern of angiogenin expression suggests a variety of functions, such as tumorigenesis, neuroprotective, antimicrobial activity, and innate immunity. Here, we comprehensively reviewed studies on the structures and functions of human and mouse angiogenins. Understanding the structure and function of angiogenins from a broader perspective could facilitate future research related to development of novel therapeutics on its biological processes, especially in gastrointestinal cancers.

## Introduction

Angiogenin is the fifth member of the vertebrate-specific RNase a superfamily firstly identified in the cell line HT-29 of human adenocarcinoma. The prime role of angiogenin is angiogenesis that is required to promote growth and metastatic spread of cancer cells. Globally, gastrointestinal cancer such as stomach, liver, and colon cancer are one of the leading causes of cancer death (Poon et al., 2003). Therefore, angiogenesis in gastrointestinal tumor is the focus of research, leading to the development of anticancer drugs. In addition to angiogenesis in gastrointestinal tumors, it has an extensive variety of capabilities, such as neuroprotective, antimicrobial activity, and innate immunity (Tello-Montoliu et al., 2006; Sheng and Xu, 2016; Prehn and Jirström, 2020; Sultana et al., 2022). Further, serum angiogenin levels are associated with different disease conditions, including cancer, cardiovascular, and inflammatory bowel diseases (Gabriel-Salazar et al., 2018; Yu et al., 2018; Garcia-Rodriguez et al., 2021). Mouse angiogenin (mAng) genes are clustered together on chromosome 14 and encode proteins with 72–81% sequence identity (Steinhelper and Field, 1992). Despite the structural similarity between human angiogenin (hANG) and mAngs, previous studies have revealed that this protein is evolutionarily different, probably connected to the versatile functions in humans and mice (Osorio et al., 2007; Codõer et al., 2010).

In this review, we discuss the structures and biological functions of hANG and murine Ang paralogs. Comparative analysis of the existing structural and functional data among angiogenin homologs provides an overview of the implications and directions for future research for the development of novel therapeutic approach, particularly in the context of gastrointestinal cancer.

## Structures of hANG and mAngs

### Structural overview

**Figure 1:**
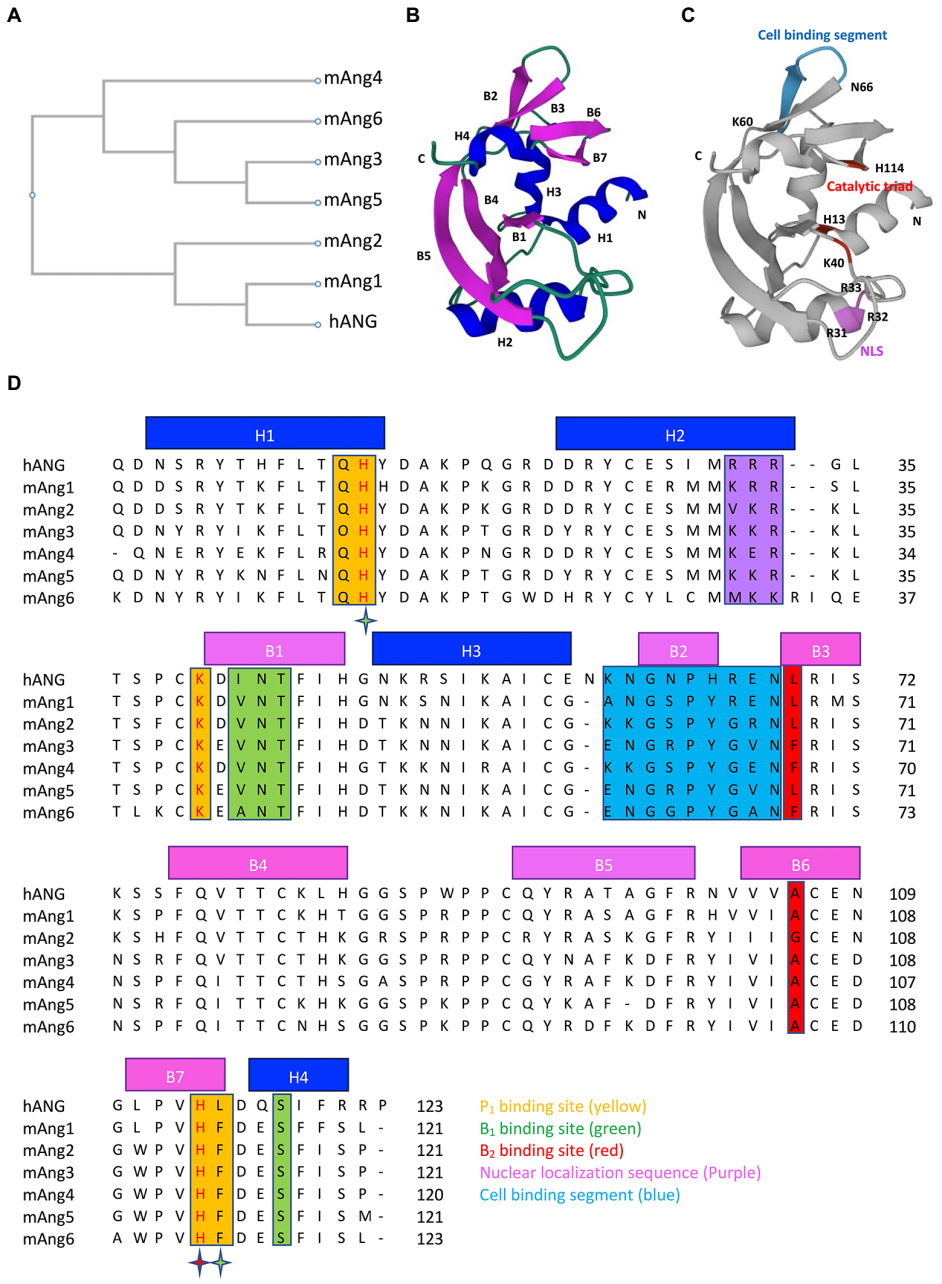
Phylogenetic tree, 3D structure and sequence alignment of angiogenins. **(A)** Phylogenetic tree of hANG and mAngs. **(B)** Three-dimensional structure of hANG (PDB entry 1B1I) labelled with secondary structures, α-helices (blue), and β-strands (purple). **(C)** Three-dimensional structure of hANG labelled with different functional sites, catalytic triad (red), NLS (purple), and cell-binding segment (blue). **(D)** Sequence alignment of the hANG and mAngs. Secondary structures are indicated by blue line (α-helices) and purple line (β-strands). Residues that form the putative substrate-binding subsites are highlighted as follows: P_1_ subsite residues (yellow), B_1_ subsite residues (green), and B_2_ subsite residues (red). The stars highlight the active site residues that belong to two subsites and are colored according to the subsite. Residues that form the NLS and cell-binding segment are highlighted in purple and blue color, respectively. The phylogenetic tree and sequences were aligned using CLUSTALW.

The sequence analysis revealed that hANG and mAng6 contain 123 amino acids; mAng1, mAng2, mAng3, and mAng5 contain 121 amino acids; and mAng4 contains 120 amino acids. The amino acid sequences of mAngs are 76.2% (mAng1), 66.4% (mAng2), 63.9% (mAng3), 62.8% (mAng4), 63.9% (mAng5), and 54.8% (mAng6) identical to those of hANG (Crabtree et al., 2007; Iyer et al., 2013). The phylogenetic tree showing the evolutionary relationship between hANG and mAngs is depicted in Figure 1A. The phylogenetic tree revealed the two major clades. The first one contains hANG, mAng1, and mAng2. The hANG gene is most closely related to the mAng1 gene, and they both have the same most recent common ancestor to the mAng2 gene. The other branches move to the different clades where the mAngs (3–6) are present. They are distinct from the hANG gene. In the angiogenin structure, the RNase A fold, comprising α-helices and β-strands connected with loop structures, is highly conserved. The structure of hANG consists of three α-helices (H1, residues 3–14 at the N terminus; H2, residues 22–33; and H3, residues 49–58), seven β-strands (B1, residues 41–47; B2, residues 62–65; B3-B4, residues 69–84; B5-B6, residues 93–108; and B7, residues 111–116), and a 3_10_ helix (residues 117–121 at the C terminus; Acharya et al., 1994; Figures 1B,D). Structural and biochemical studies have established that angiogenin has three distinct functional sites: (i) catalytic triad, (ii) nuclear localization sequence, and (iii) cell-binding site. In addition, studies on angiogenin structure have highlighted other amino acid residues that are essential for its biological activity in the P_1_, B_1_, and B_2_ subsites (Curran et al., 1993; Russo et al., 1996; Holloway et al., 2005). Residues present in the P_1_, B_1_, and B_2_ subsites of mAngs occupy similar positions to hANG counterparts and are supposed to have comparable activities (Figures 1C,D).

### Catalytic site

All members of RNase A superfamily contain catalytic sites which specifically cleave tRNA on the 3′-side of the pyrimidine nucleotides. Pyrimidine bases preferentially bind to the B_1_ subsite, whereas the nearby scissile phosphodiester linkage binds to P_1_ and the purine bases bind to the B_2_ subsite (Parés et al., 1991). In the case of hANG, catalytic triad is present in the P_1_ subsite, comprising His13, Lys40, and His114. The RNase activity of hANG was 10^4^–10^6^ fold less than that of RNase A (Shapiro et al., 1986) and this weakness of RNase activity of hANG is due to differences in structural features such as pyrimidine binding site obstruction by Gln 117, hydrogen bond between Thr44 and Thr80 that suppresses the activity of the pyrimidine site, and the absence of a structural counterpart important for catalysis (Leonidas et al., 1999). Importantly, the catalytic triad residues were conserved in all members of angiogenin (Figures 1C,D). Angiogenins exhibit structural similarity at the catalytic site, with some variations in their enzymatic activities. In comparison to hANG, all mAngs show reduced RNase catalytic activity because the hydrophobic interactions in between C-terminal segment and the main body of the protein seem to be more intense than those in hANG (Holloway et al., 2005). Paralogs mAng1, mAng3, and mAng4 have similar activities; however, mAng2 is more effective at cleaving tRNA. The possible reason is the lack of intermolecular interactions in mAng2 that may stabilize the C-terminal catalytic residue His113, which represents a more open active site than other paralogs (Iyer et al., 2013). These observations demonstrate that the different catalytic activities of angiogenin are due to the variation in important amino acid positions, which changes the intermolecular interactions in the structure of proteins.

### Nuclear localization sequence

The amino acid residues in the NLS of both hANG and mAngs are presented in Figures 1C,D. Three consecutive cationic residues Arg31, Arg32, and Arg33 comprise the NLS of hANG of which Arg33 is crucial for nuclear targeting, whereas Arg31 and Arg32 play modulatory roles (Moroianu and Riordan, 1994). The NLS sequence of mAng1 (Lys 31, Arg 32, and Arg 33) and mAng3 (Lys 31, Lys 32, and Arg33) consists of three basic residues, but substitutions of Arg31 to Lys 31 for mAng1 and Arg31 to Lys31, and Arg32 to Lys32 for mAng3 from hANG have been observed (Iyer et al., 2013). However, this substitution did not alter the surface charge distribution. Furthermore, mAng5 retains the NLS (Lys 31, Lys 32, and Arg33) in the same manner as mAng3 (Iyer et al., 2013). Although the essential arginine (Arg33) is conserved in mAng2 (Val31, Lys32, and Arg33), substitution of Arg31 with Val31 alters the NLS surface charge (Iyer et al., 2013). The corresponding segment of mAng4 has a different sequence: Lys30, Glu31, and Arg32. The backbone conformation and side-chain positions of mAng4 and hANG are well conserved in this region, but the surface charge distribution differs because of the substitution of Glu31 for Arg32. Despite the consecutive absence of positive charges in the mAng4 structure, novel substitutions of amino acid residues in the vicinity of the mAng4 structure from hANG, including Lys7, Arg10, and Lys33, instead of His8, Thr11, and Gly34, provided extra positive charges that are responsible for the abundance of cationic residues on the surface of mAng4, which contribute to NLS (Jans et al., 2000; Crabtree et al., 2007).

### Cell-binding site

The positions of cell-binding segments in hANG and mAngs are shown in Figures 1C,D. Amino acid residues 60–68 (KNGNPHREN) and Asn109 of hANG are known to be involved in cell-binding (Hallahan et al., 1991, 1992). Mutagenesis studies have highlighted that the residues Asn61 and Arg66 in the cell-binding site are important for the angiogenic activity of hANG (Hallahan et al., 1991, 1992). In mAng1, the important residues of the cell-binding segment, whose sequence is ANGSPYREN, are well conserved in hANG but vary more in other mAngs (Holloway et al., 2005). The mAng2 cell-binding sequence (KRGSPYGRN) has critical amino acid substitutions Asn → Lys at position 60 and Arg → Gly at position 65, which are counterparts of Asn61 and Arg66 of hANG (Iyer et al., 2013). Although mAng2 has structural similarities with other mAngs, its function shifted to non-angiogenic due to differences in residues in the cell-binding segment, as compared to mAng1. Arg66 of hANG, which is critical for angiogenic activity, is replaced by Gly residue (position 65) in the sequence of mAng3 (ENGRPYGVN; Iyer et al., 2013). In case of mAng4 cell-binding segment (KKGSPYGEN), the crucial residues of hANG, Asn61 and Arg66, are replaced with Lys59 and Gly64, respectively (Crabtree et al., 2007). However, Lys59 in mAng4 seems to be crucial for angiogenic activity, since the substitution of Lys59 → Asn59 by mutation abolished angiogenic activity (Crabtree et al., 2007). In addition, structural modification of the cell-binding segment has no appreciable effect on RNase activity (Crabtree et al., 2007; Sultana et al., 2022).

## Biological functions of angiogenins in humans and mice

A summary of the biological functions of hANG and mAngs is presented in Table 1.

**Table 1:** Summary of biological functions of human and mouse angiogenins.

Name	Biological functions	Description	References
hANG	Antimicrobial and antiviral	Exhibits antimicrobial activity against *Streptococcus pneumoniae*, *Enterococcus faecalis*, *Listeria monocytogenes*, *Candida albicans*, *Klebsiella pneumoniae*, *Mycobacterium tuberculosis*, *Pseudomonas aeruginosa*, *Escherichia coli*, and HIV-1.	Hooper et al. (2003), Bedoya et al. (2006), Cocchi et al. (2012), and Noschka et al. (2021)
	Wound healing and tissue regeneration	hANG promotes wound healing and tissue regeneration by activating tissue plasminogen activator, fibroblast cells, PI3K signaling, and angiogenesis.	Hu and Riordan (1993), Dutta et al. (2014), Cucci et al. (2021), Yurina et al. (2021)
	Skeletal growth	hANG is important for skeletal growth *via* activation of the angiogenin/plexin b2 ribosomal biogenesis signaling pathway.	Liu et al. (2021)
	Neuroprotective	hANG is able to extend neurite and survival of P19 EC cell derived motor neurons from hypoxia and also improves motor function and extends lifespan in ALS mouse model.	Kieran et al. (2008), Subramanian et al. (2008), Aparicio-Erriu and Prehn (2012), Prehn and Jirström (2020)
	Metabolism of cytoplasmic tRNA	Metabolism of cytoplasmic tRNA by hANG are crucial for the gastrointestinal cancer cell survival, development and progression.	Razavi et al. (2021), Akiyama et al. (2022)
	Tumorigenesis	hANG is involved for the growth of tumor and metastasis in many cancer types including pancreatic and prostate. The mechanistic insight of tumor growth by hANG is the expression of matrix metallopeptidase 2 *via* the ERK1/2 pathway.	Poon et al. (2003), Miyake et al. (2015), Vanli and Guo-Fu (2015), Wang et al. (2018)
	Diagnostic biomarker	hANG may serve as diagnostic biomarker for several diseases such as gastrointestinal cancers, cardiovascular, and inflammatory bowel diseases.	Gabriel-Salazar et al. (2018), Yu et al. (2018), Garcia-Rodriguez et al. (2021)
	Angiogenesis	hANG promotes transcription of rRNA and mRNA, as well as activates various signaling pathways such as ERK1/2, PI3K/Akt, SAPK/INK enabling various functions such as cell proliferation, migration, invasion, survival, and tube formation.	Liu et al. (2001), Xu et al. (2001), Tsuji et al. (2005), Yoshioka et al. (2006), Trouillon et al. (2010), Hoang and Raines (2017), Yu et al. (2017)
mAng1	Angiogenic	mAng1 exhibits angiogenic activity on chick chorioallantoic membrane and induce sprouting from thoracic aorta.	Nobile et al. (1996), Crabtree et al. (2007)
	Antimicrobial	mAng1 exhibits potent antifungal and antibacterial against *Candida albicans* and *Streptococcus pneumoniae*, respectively.	Hooper et al. (2003)
	Neuroprotective	mAng1 displays a neuroprotective role in Parkinson’s disease by protecting against neuronal cell death induced by rotenone and neurotoxins 1-methyl-4-phenylpyridinium through the PI3K-Akt signaling pathway.	Steidinger et al. (2011)
	Anti-inflammatory	mAng1 regulates gut microbiota composition evidenced by mAng1-knockout mice leading to severe colitis by increasing the colitogenic strains of *α-Proteobacteria* and decreasing the protective gut commensal strains of Lachnospiraceae.	Sun et al. (2021)
mAng2	Non-angiogenic	mAng2 exhibits non-angiogenic activity due to its inability to bind cellular receptor.	Nobile et al. (1996), Crabtree et al. (2007), Iyer et al. (2013)
mAng3	Angiogenic	mAng3 exhibits angiogenic activity in rat cremaster muscle and chicken embryo chorioallantoic membrane. In endothelial cell, mAng3 increase mitochondria, polysomes, and endoplasmic reticulum.	Fu et al. (1999), Iyer et al. (2013)
mAng4	Angiogenic	mAng4 demonstrates angiogenic activity in the thoracic aorta.	Crabtree et al. (2007)
	Antibacterial	mAng4 demonstrates antibacterial effects against *Bacteroides thetaiotaomicr*on, *Enterococcus faecalis*, *Bifidobacterium longum*, *Enterococcus gallinarum*, *Salmonella typhimurium* SL1344, *Listeria monocytogenes*, and *Salmonella typhimurium* LT2.	Hooper et al. (2003), Walker et al. (2013), Sultana et al. (2022)
	Worm expulsion	mAng4 expression is associated with worm clearance such as *Trichuris muris* and *Trichinella spiralis* by T_H_2 cytokine-dependent immune response.	Datta et al. (2005), Angkasekwinai et al. (2013), Noor et al. (2016)
	Gut microbiota homeostasis	mAng4 maintains gut microbiota homeostasis by increasing good bacteria including *Akkermansia*, *Lactobacillus*, *Dubosiell*a, *Adlercreutzia*, and *Coriobacteriaceae* UCG-002, and reducing certain harmful bacteria, such as *Alistipes* and *Enterorhabdus*.	Sultana et al. (2022)

### hANG

hANG is expressed in gastrointestinal adenocarcinomas, colonic epithelial tumor cell lines, immune, epithelial, and endothelial cells, as well as in blood plasma (Wu et al., 2007; Schwartz et al., 2018). The comprehensive expression characters of hANG suggest that the physiological function is not restricted to the neovascularization process (Moenner et al., 1994).

In colorectal and pancreatic cancer, elevated hANG expression is linked to higher tumor microvessel density and lower patient survival (Vanli and Guo-Fu, 2015; Wang et al., 2018). hANG is involved in tumor angiogenesis in gastrointestinal cancers. In tumors, the formation of new blood vessels is a multistage process. After releasing from the tumor cells, hANG binds to specific endothelial cell receptors in preexisting blood vessels, activating the endothelial cells to release enzymes that break down the basement membrane. The newly formed capillary tubes are then formed by the proliferation, migration, and assembly of the activated endothelial cells. Thereafter, a new basement membrane is produced, the vessels mature, and a vascular lumen is formed. In addition, hANG triggers the protease cascades that facilitate the migration of cancer cells through the extracellular matrix (Poon et al., 2003).

The molecular mechanism of angiogenesis induced by hANG has to be discussed. The sequential steps to induce angiogenesis by hANG are initiated by binding of hANG to hANG receptors (170 kDa cell surface protein or plexin b2 receptor). Then, hANG is incorporated into the endothelial cell by endocytosis and rapidly translocated to the nucleus following to mediate several intracellular pathways such as PKB/Akt, ERK1/2, and SAPK/JNK. This signaling activation promotes ribosomal RNA transcription which acts as a key step in ribosome biogenesis and drives the angiogenesis accompanied by upregulation of gene sets related to cell proliferation, migration, invasion, and tube formation (Tsuji et al., 2005; Hoang and Raines, 2017; Yu et al., 2017).

hANG plays a significant role for the metabolism of cytoplasmic tRNA. Recent research has shown that in response to stress conditions, such as oxidative, starvation, and hypoxia, hANG cleaves the conserved single-stranded 3′-CCA termini of tRNA or anticodon loop of tRNA to form tiRNA (tRNA-derived, stress-induced small RNA), which inhibit protein synthesis, activate cytoprotective stress response programs, and promote cell survival resulting in progression of gastrointestinal cancers (Yamasaki et al., 2009; Ivanov et al., 2011; Li and Hu, 2012; Lyons et al., 2017; Razavi et al., 2021; Akiyama et al., 2022). The molecular insights of hANG to improve cell survival in stressful conditions are mediated by tiRNA, which selectively facilitates translation of anti-apoptosis genes and decreases the generation of apoptosome proteins produced during apoptosis and thus promotes cell survival (Goncalves et al., 2016; Akiyama et al., 2022). Collectively, metabolism of cytoplasmic tRNA by hANG is crucial for the gastrointestinal cancer cell survival, development, and progression. Therefore, this process may also serve as targets for cancer therapy (Razavi et al., 2021).

The function of hANG in case of other diseases has been reported. Loss of functions mutations in the *hANG* gene has been detected in neurodegenerative disorders such as amyotrophic lateral sclerosis (ALS) as well as Parkinson’s disease (Greenway et al., 2006; Van Es et al., 2011; Aparicio-Erriu and Prehn, 2012; Fasoli et al., 2021). Functional assays showed that these mutations result in complete loss of function by disabling angiogenesis because of deficiency in RNase activity, nuclear translocation, or both (Wu et al., 2007). Administration of hANG to an ALS mouse model improves motor function and extends lifespan (Kieran et al., 2008). To exert neuroprotective functions, hANG shows dual actions on both motoneurons and astrocytes *via* the protein synthesis reprogramming. The underlying mechanism is that hANG binds syndecan (receptor of hANG) to be uptaken into the astroglia and cleaves the tRNA by its ribonuclease activity to produce tiRNA which plays an important role for protein synthesis reprogramming. As a result, the protein translation profile is altered, and the motoneurons receive a survival signal *via* the PI3K/Akt pathway (Aparicio-Erriu and Prehn, 2012; Prehn and Jirström, 2020).

hANG is also known to have an important contribution in tissue regeneration and wound healing as a part of the response to injury. hANG interacts with actin and forms a complex that activates tissue plasminogen activator (tPA) to generate plasmin inducing basement membrane and extracellular matrix degradation that characterizes the processes of wound healing and repair (Hu and Riordan, 1993; Dutta et al., 2014). In addition, hANG activates fibroblast cells to produce extracellular matrix proteins such as fibrin, collagen, and fibronectin to facilitate wound healing (Cucci et al., 2021; Yurina et al., 2021).

Osteoclast-derived angiogenin is important for skeletal growth *via* activation of the angiogenin/plexin b2 ribosomal biogenesis signaling pathway. Obstruction of this pathway by glucocorticoids (GCs) causes senescence of vascular cells, and results in impairment of angiogenesis and osteogenesis. Administration of recombinant hANG, which antagonizes cellular senescence in vascular endothelial cells, reduces the negative effects of GCs on the developing skeleton (Liu et al., 2021).

In addition, hANG exhibits antimicrobial activity against *Streptococcus pneumoniae*, *Enterococcus faecalis*, *Candida albicans*, and *Listeria monocytogenes* (Hooper et al., 2003). The antibacterial activity is mediated by the binding to anionic surfaces of bacterial membranes, forming pores, and penetrating into the phospholipid bilayer of bacteria through its amphipathic structure in hydrophobic environment (Schwartz et al., 2018). In case of viral infection, the antiviral activity of hANG against human immunodeficiency virus-1 (HIV-1) has been reported. To exert antiviral activity, hANG enters the virus-infected cells by pinocytosis that facilitates the interaction of hANG and virions to degrade viral nucleic acid resulting inhibition of HIV-1 replication (Bedoya et al., 2006). Further research revealed the activity of hANG against X4 strains of HIV-1 in which hANG is produced from the primary T cells at the concentration effective for antiviral activity and appears to be a major factor of anti-X4 activity (Cocchi et al., 2012).

### mAng1

mAng1 is abundantly expressed in the liver, with smaller extent in the pancreas and lungs (Hooper et al., 2003). mAng1 regulates gut microbiota composition evidenced by mAng1-knockout mice leading to severe colitis by increasing the colitogenic strains of *α-Proteobacteria* and decreasing the protective gut commensal strains of *Lachnospiraceae* (Sun et al., 2021). In addition, mAng1 exhibits potent antifungal and antibacterial activities agains*t Candida albicans* and *Streptococcus pneumoniae*, respectively (Hooper et al., 2003). Moreover, mAng1 displays a neuroprotective role in Parkinson’s disease by protecting against neuronal cell death induced by rotenone and neurotoxins 1-methyl-4-phenylpyridinium through the PI3K-Akt signaling pathway (Steidinger et al., 2011).

### mAng2

mAng2, also known as angiogenin-related protein (Angrp), shares 78% sequence identity with mAng1 (Brown et al., 1995; Nobile et al., 1996). The RNase activity of mAng2 toward dinucleotide substrates as well as tRNA is greater than that of other mAngs (Iyer et al., 2013). However, mAng2 appears to be non-angiogenic, as estimated by the chick chorioallantoic membrane angiogenesis assay, where the number of blood vessel branch points is counted for the quantification of blood vessels (Nobile et al., 1996). Moreover, its inability to bind to cellular receptors due to substitution of the important residues, Asn61 and Arg66, in the cell-binding site might be a possible reason for the lack of angiogenic activity (Nobile et al., 1996).

### mAng3

mAng3 is firstly discovered by transcriptional activation in NIH3T3 cells transformed with the E2a-Pbx1 oncoprotein (Fu and Kamps, 1997). Expression of mAng3 was found in lung tissues and adult prostate (Hooper et al., 2003). mAng3 has angiogenic activity, as evidenced by the angiogenesis in the rat cremaster muscle and chicken embryo chorioallantoic membrane. Electron microscopic image revealed that endothelial cells in mAng3 induced vessels possessed fenestrations comparable to those found in endothelial cells from neovasculature induced by growth factor of vascular endothelial and basic fibroblast. Furthermore, mAng3 causes remarkable molecular changes in rapidly proliferating endothelial cells, such as increasing the mitochondria, polysomes, and endoplasmic reticulum (Fu et al., 1999).

### mAng4

mAng4 is expressed in the small intestine by Paneth and goblet cells and is secreted into the gut lumen together with other secretory contents, such as lysozyme (Hooper et al., 2003; Forman et al., 2012; Walker et al., 2013). mAng4 expression in the small intestine is upregulated by colonization of *Bacteroides thetaiotaomicron*. In addition, the expression of mAng4 was detected in conventionally raised mice but not in germ-free mice, suggesting that gut bacteria are responsible for greater mAng4 expression (Hooper et al., 2003). Furthermore, mAng4 expression is associated with worm clearance by T_H_2 cytokine-dependent immune response (Datta et al., 2005; Angkasekwinai et al., 2013).

mAng4 demonstrates antibacterial effects against *Bacteroides thetaiotaomicron, Enterococcus faecalis, Bifidobacterium longum, Enterococcus gallinarum, Listeria monocytogenes*, and *Salmonella typhimurium* LT2 (Hooper et al., 2003; Walker et al., 2013). Recent studies showed that disruption of bacterial membrane is needed to kill bacteria by mAng4 (Sultana et al., 2022).

In addition, mAng4 has been attributed to angiogenesis, which is determined by the evaluation of *ex vivo* angiogenesis through thoracic aorta assay (Crabtree et al., 2007). Mutational studies have revealed that ribonucleolytic activity is essential for angiogenic activity, as assessed by using H12A and H112A mutants (Crabtree et al., 2007). Moreover, residues present in the cell-binding segment, K59, and nuclear localization sequence, R32, play crucial roles in angiogenesis (Crabtree et al., 2007).

## Future perspective

Currently, the clinical application of angiogenin has not been established. However, increasing evidence suggests that it may be helpful in the diagnosis and prognosis prediction of several diseases. Variations in serum angiogenin levels in different diseases, including cancer, cardiovascular and inflammatory bowel diseases, suggest that angiogenin is a potential disease diagnostic marker candidate (Yu et al., 2018). However, there is a scarcity of clinical trial data, and further research on its prospective clinical applications is required.

Over the past 10 years, numerous anti-angiogenic therapies have been developed for the treatment of gastrointestinal cancer, and at least 80 medications are currently being tested in preclinical research and phase I–III clinical trials. However, this anti-angiogenic therapy imparted resistance due to the activation of alternative pathways that maintain tumor growth and vascularization (Haibe et al., 2020). On the other hand, the functional sites of angiogenins such as RNase catalytic sites, nuclear localization sequences, and cell-binding segments are involved in angiogenic activity. Thus, inhibitor of these functional sites could be therapeutic agents for angiogenin-mediated angiogenesis. In addition, therapies that antagonize angiogenin activity by disrupting angiogenin binding to its receptors (170 kDa cell surface protein or plexin b2) might also be a promising therapeutic approach for treating cancer (Yoshioka et al., 2006; Yu et al., 2017). Combination of anti-angiogenic drugs and hANG inhibitor might lead to better therapeutic outcome of gastrointestinal cancers. Because clinical therapy of anti-angiogenic drugs focused on the inhibition VEGF or tyrosine kinases, combination therapy might block the tumor-driven angiogenesis mediated by hANG and other angiogenic factors.

In neuroprotective treatments, recombinant angiogenin has been reported to significantly reduce mortality in mice model of ALS and Parkinson’s diseases (Kieran et al., 2008; Steidinger et al., 2011). Therefore, it is also important to focus on the findings of clinical trials of angiogenin in neurodegenerative disorders.

It is reported that angiogenin is effective to kill highly pathogenic bacteria such as *Klebsiella pneumoniae* and *Pseudomonas aeruginosa* (Noschka et al., 2021). The antibacterial action of angiogenin is accomplished by disrupting the cell membrane of bacteria, in contrast to conventional antibiotics that hinder cell wall synthesis, DNA replication, RNA transcription, and protein synthesis (Sultana et al., 2022). Thus, angiogenin may not be prone to the rapid development of drug resistance due to different killing mechanism against bacteria. In addition, angiogenin will be therapeutic targets for the anti-HIV activity.

The 3D structures and functional sites of hANG and mAngs are remarkably similar and are hypothesized to exhibit comparable activities. Additionally, it has been discovered that mAngs and hANG share similar physiological roles, such as angiogenesis, tumorigenesis, neuroprotection, antibacterial effects, and innate immunity (Sheng and Xu, 2016). Recent research has shown that hANG may serve as a therapeutic target as well as a diagnostic biomarker for gastrointestinal cancers (Yu et al., 2018). Therefore, regarding the structural and functional similarity between mAngs and hANG, our findings strongly suggest that comprehensive studies of mAngs will provide information that may facilitate the identification of therapeutic targets against gastrointestinal cancers in humans.

## Conclusion

In conclusion, the multifunctional protein angiogenin may serve as an excellent clinical target, and diagnostic marker. Since tumor angiogenesis is responsible for gastrointestinal tumor growth, metastasis, and survival, the simultaneous inhibition of tumor-driven angiogenesis mediated by angiogenins and vascular endothelial growth factors would represent one of the major therapeutic approach against cancer. Extensive research has been reported on hANG, whereas studies on mAngs in relation to its biological activities are limited. The relationship between the structure and function of angiogenins was covered in this review. With the development of new technologies, further roles of angiogenin are expected to be identified in the near future.

## Author contributions

MFS, HA, and HK conceived the idea for this review. The review was initially written by MFS and was critically edited by HA and HK. All authors contributed to the article and approved the submitted version.

## Funding

This study was supported in part by the Japan Agency for Medical Research and Development (AMED) under grant numbers 22ae0121017h0002 and 22ym0126065h0001, and the Institute for Advanced Academic Research of Chiba University (to HK).

## Conflict of interest

The authors declare that the research was conducted in the absence of any commercial or financial relationships that could be construed as a potential conflict of interest.

## Publisher’s note

All claims expressed in this article are solely those of the authors and do not necessarily represent those of their affiliated organizations, or those of the publisher, the editors and the reviewers. Any product that may be evaluated in this article, or claim that may be made by its manufacturer, is not guaranteed or endorsed by the publisher.
